# Microbroth dilution method for antibiotic susceptibility testing of fastidious and anaerobic bacteria of the urinary microbiome

**DOI:** 10.1128/spectrum.00314-24

**Published:** 2024-05-06

**Authors:** Wilson Geaman, Brian I. Choi, Jacob Kaindl, Caroline Gonzalez, Alan J. Wolfe

**Affiliations:** 1 Department of Microbiology and Immunology, Loyola University Chicago, Maywood, Illinois, USA; University of Exeter, Exeter, United Kingdom

**Keywords:** urinary tract infection, fastidious isolates, anaerobes, antibiotic resistance, susceptibility testing, human microbiome, microbiome

## Abstract

Bacterial isolates from the human urinary microbiome have been extensively studied for their antibiotic resistance; however, little work has been done on those isolates that are difficult to grow *in vitro*. This study was designed to qualify a serum-based medium, New York City Broth III (NYCIII), and a broth microdilution method to determine the antibiotic susceptibility of previously underreported or undescribed microbes that have a difficult time growing in standard Mueller-Hinton broth. Here, we demonstrate that NYCIII microbroth dilution can be an effective method for the determination of antibiotic susceptibility of species found in the human urinary microbiome. We show that this method serves well to characterize fastidious and anaerobic urinary microbes that have no Clinical and Laboratory Standards Institute (CLSI) guidelines, including several in the families *Aerococcaceae*, *Lactobacillaceae*, or *Actinomycetaceae*. Previous studies using expanded quantitative urine culture reveal that urine samples from clinical patients are commonly polymicrobial in composition. Thus, we test whether NYCIII can serve as a viable harmonized medium, capable of supporting antibiotic susceptibility testing in a range of fastidious, non-fastidious, and anaerobic urinary microbes. We propose this methodology to be standardized comparable to CLSI standards to allow for resistance testing in uncharacterized urinary bacteria.

**IMPORTANCE:**

Antibiotic susceptibilities of fastidious and anaerobic bacteria of the human urinary microbiome are largely underreported due to difficulty in growing them in the lab environment. The current standard medium, Muller-Hinton broth, has difficulty supporting the growth of many of these species, leaving microbiologists without a standardized method. To address this need, this study offers a methodology to survey susceptibilities in a high-throughput manner of these understudied microbes with a proposed harmonized medium, NYCIII, which is capable of supporting the growth of both fastidious and non-fastidious urinary microbes. Broader standardization of this method can allow for the development of antibiotic-resistant breakpoints of the many uncharacterized urinary microbes.

## INTRODUCTION

Antibiotic susceptibility testing (AST) of bacteria of the urinary tract is a vital tool in guiding proper therapy toward treating infection; however, this practice is primarily limited to a handful of selected species labeled as uropathogens. This designation is most often reserved for certain Gram-negative species, such as *Escherichia coli*, *Pseudomonas aeruginosa*, *Klebsiella pneumoniae*, and *Proteus mirabilis*, and certain species of the Gram-positive genera *Enterococcus*, *Staphylococcus*, and *Streptococcus* (1–3). With plenty of evidence implicating these species in lower urinary tract disorders, such as urinary tract infections, there are sophisticated guidelines and standards for evaluating AST, as outlined by the Clinical and Laboratory Standards Institute (CLSI) and the European Committee on Antimicrobial Susceptibility Testing (EUCAST) (4–6).

The standard urine culturing methods designed to detect the most common uropathogens do not support the growth of fastidious and anaerobic bacteria (7). The resulting bias has led to underreporting and thus understudy of such species. Many of these species are now considered to be “emerging” uropathogens, a recognition made possible by recent advances in metagenomics and metaculturomics technologies (8–11). Such methods include those such as expanded quantitative urine culture (EQUC) coupled with matrix-assisted laser desorption/ionization-time of flight mass spectrometry (MALDI-TOF MS) (12–14). Unfortunately, apart from the limited number of species listed by CLSI in M45-ED3:2016, there exist few standards or guidelines for the evaluation of AST in uropathogens (4).

Only in 2016 CLSI first published guidance and clinical breakpoints for several fastidious urine microbes, such as *Aerococcus*, but it has yet to further add to or expand upon them. As the growth medium for broth microdilution, CLSI recommends cation-adjusted Mueller-Hinton broth supplemented with lysed horse blood (CAMHB-LHB) (2.5% to 5% vol/vol) with incubation at 35°C with 5% supplemented CO_2_ for 24 to 48 hours (5). Similarly, EUCAST recommendations for *Aerococcus* is Mueller-Hinton broth supplemented with 5% lysed horse blood and 20 mg/L β-NAD (MH-F broth) with incubation at 35°C ± 1°C in ambient air for 18 ± 2 hours (6). The common theme is the supplementation of the standard medium with lysed horse blood, which has been previously utilized to improve the growth of other fastidious microbes, such as *Corynebacterium diphtheriae* and *Haemophilus influenzae* (15, 16). Although effective in promoting growth, the use of blood renders the media opaque and thus prevents the measurement of bacterial growth via optical density. To conduct AST on many strains, we sought a medium that could (i) support the growth of fastidious and anaerobic microbes and (ii) allow direct monitoring of bacterial response to antibiotics.

In evaluating media, a significant candidate is New York City Broth III (NYCIII). This medium was originally developed to promote the growth and isolation of fastidious *Neisseria* species from clinical specimens (17, 18). Although now fallen out of favor compared with the use of Thayer-Martin media, NYCIII is still remarkable for its transparency and, consequently, its ability to support turbidity measurements via spectrometry. This property is attributed to the use of horse serum instead of lysed horse blood. Taking advantage of this ability, one study found that NYCIII was the most ideal of nine different media compositions to promote the growth and measurement of bacterial vaginosis-associated anaerobes (19). Looking to extend these findings, we evaluated NYCIII for evaluation of AST in fastidious and anaerobic species of the urinary microbiome. Herein, we investigate the use of this methodology alongside CLSI standards and evaluate AST in previously untested urinary microbes. We also expand this method to include testing in co-cultures to evaluate interactions between species that may increase or decrease resistance to certain antibiotics when grown together.

## MATERIALS AND METHODS

### Study isolates

Standardized reference strains for quality control (QC) were selected as outlined by CLSI and were procured from the American Type Culture Collection (ATCC) (4, 5). Urinary isolates for resistance testing were procured from our IRB-approved biorepository (LU 215192). A complete list of strains used in this study can be found in Table S1. Each strain tested was grown from frozen stock, either purchased from ATCC or frozen in brucella media at −80°C. Strains were struck out on trypticase soy agar (TSA) with 5% sheep blood, and identity was confirmed via MALDI-TOF MS.

### Antibiotic and media preparation

Preparations of stock antibiotics were as outlined in CLSI M100-ED33:2023. Preparation of CAMHB-LHB broth was according to CLSI instructions. NYCIII broth was prepared as outlined by ATCC medium #1685 with modification: 4.2 mL HEPES buffer solution, 7.5 g proteose peptone no. 3, 2.5 g glucose, 2.5 g NaCl, and 1.875 g yeast extract were added to 450 mL Millipore water before autoclaving at 121°C for 15 minutes. After cooling, 25 mL heat-deactivated horse serum and 25 mL heat-deactivated newborn calf serum were added via vacuum filter filtration (0.2 μm) to form the final 500 mL solution. For culturing anaerobic bacteria, the media were conditioned in an anaerobic chamber overnight. A comparison of the ingredients of CAMHB-LHB versus NYCIII can be found in Table 1. A complete list of brand components can be found in Table S2. To compare the growth of microbes between the two different media, single colonies struck on TSA plates were inoculated into 5 mL of test media and allowed to incubate for 48 hours (72 hours for anaerobic species). Afterward, cultures were evaluated for growth via colony-forming unit per milliliter (CFU/mL) enumeration.

**TABLE 1:** Composition of CAMHB-LHB versus NYCIII

CAMHB-LHB		NYCIII	
Beef extract	3 g	Proteose peptone no. 3	15 g
Acid hydrolysate of casein	17.5 g	Yeast extract	3.75 g
Lysed horse blood	5% vol/vol	Heat-inactivated horse serum	50 mL
		Heat-inactivated neonatal bovine calf serum	50 mL
Starch	1.5 g	Glucose	5 g
MgCl_2_·6H_2_O	10 mg	HEPES (1 M)	4 mL
CaCl_2_·2H_2_O	20 mg	NaCl	5 g
H_2_O	Adjust to 1 L	H_2_O	Adjust to 1 L

### Susceptibility testing

Strains were started in tubes with 5 mL of the test growth media until mid-log phase at 37°C with 5% supplemented CO_2_ and shaking at 200 rpm. For the microbroth dilution method, 20 µL of this inoculum was added to 180 µL of fresh test media with or without antibiotics within the wells of a flat-bottom transparent 96-well plate. This plate was placed within a BioTek Epoch 2 Microplate Spectrophotometer, which incubated the bacteria at 37°C with 5% supplemented CO_2_ and shaking at 200 rpm (double orbital). Absorbance was read every 15 minutes at 600 nm for 72 hours. Growth curves were analyzed to determine the minimal inhibitory concentration (MIC). For quality control testing, antibiotic dilutions were made via the standard twofold dilutions. For exploring susceptibility ranges in the fastidious and anaerobic urinary strains, antibiotic dilutions were conducted in 10-fold dilutions to capture a wider range of possible antibiotic susceptibility in unknown, undomesticated strains. All experiments were conducted in duplicate.

## RESULTS

### Comparison of standard media with NYCIII

**Fig 1:**
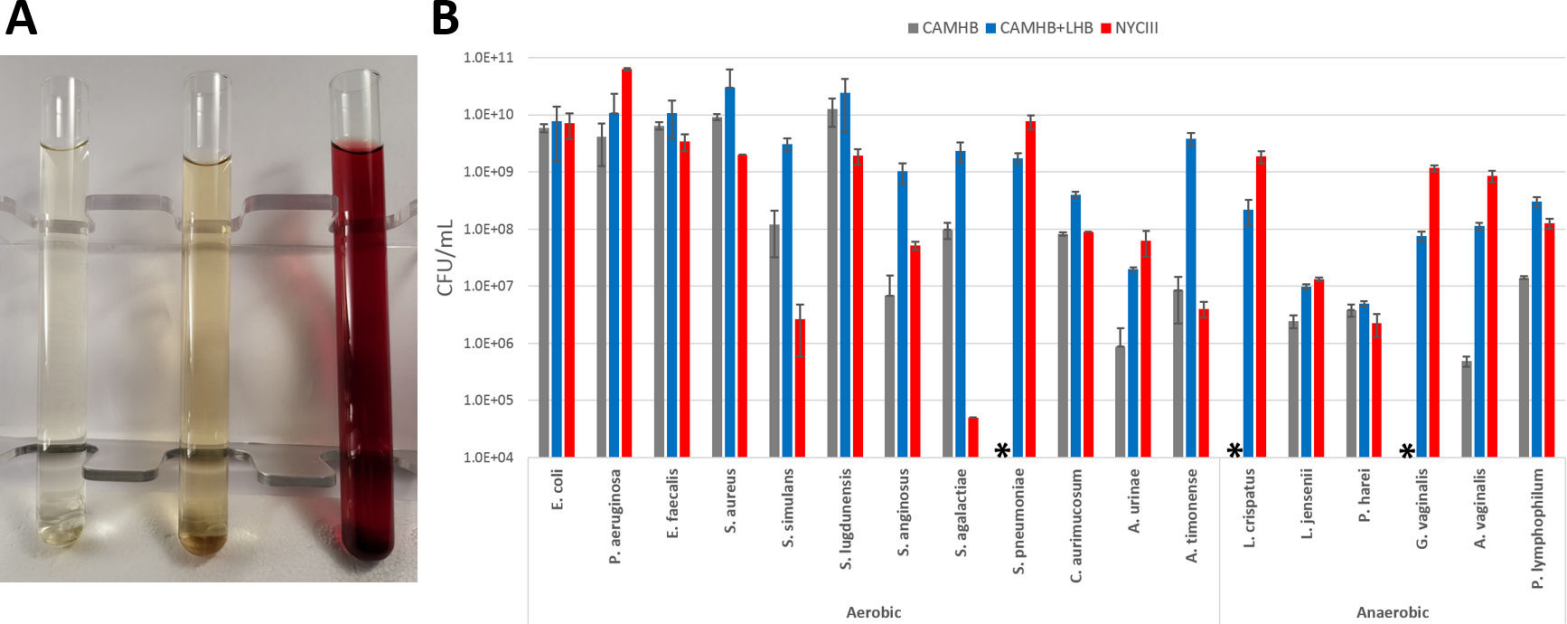
NYCIII compared to standard media. (A) Visual comparison of media. Left to right: CAMHB, NYCIII, CAMHB-LHB. (B) Growth comparison of representative strains. Aerobic conditions: 24-hour incubation for non-fastidious species, 48-hour incubation for fastidious species at 37°C with 5% supplemented CO_2_ with shaking. Anaerobic conditions: 72-hour incubation at 37°C with shaking. Experiments conducted in triplicate. Strains tested: *E. coli* ATCC25922, *P. aeruginosa* ATCC27853, *E. faecalis* ATCC29212, *S. aureus* ATCC29213, *S. simulans* UMB10066, *S. lugdunensis* UMB12897, *S. anginosus* UMB8616, *S. agalactiae* UMB11507, *S. pneumoniae* ATCC49619, *C. aurimucosum* UMB7398, *A. urinae* ATCC51628, *A. timonense* UMB8037, *L. crispatus* UMB0821, *L. jensenii* UMB7766, *P. harei* UMB0065, *G. vaginalis* UMB0540, *A. vaginalis* UMB0204, and *P. lymphophilum* UMB11620. Asterisk (*) indicates no growth for the given medium.

Although CAMHB can support the growth of common uropathogens, it does a poor job of supporting the rarer fastidious urinary microbes (Fig. 1). For example, no growth is observed at all for the pathogen *Streptococcus pneumoniae* and the commensal *Lactobacillus crispatus* for this medium. Upon supplementation with lysed horse blood, the fastidious microbes have enhanced growth. Similar growth is maintained in NYCIII compared to CAMHB-LHB for most common uropathogens. Importantly, growth is strongly observed in NYCIII for the species that demonstrated no growth in CAMHB.

### Validation of AST method

Furthering our evaluation of NYCIII, we next sought a methodology to conduct AST on a large number of isolate-antibiotic combinations utilizing spectrometry. This method is based on the twofold broth microdilution method as outlined by CLSI with modification to be conducted in a 96-well format. The output of bacterial growth curves when grown in the presence of increasing concentrations of antibiotic was analyzed to determine the MIC. This method was first applied to the non-fastidious microbes *Staphylococcus aureus* ATCC29213, *Enterococcus faecalis* ATCC29212, *Escherichia coli* ATCC25922, and *Pseudomonas aeruginosa* ATCC27853 grown in CAMHB at 37°C in ambient air with shaking and the fastidious microbe *S. pneumoniae* ATCC49619 grown in CAMHB with 5% horse serum (vol/vol) at 37°C with 5% supplemented CO_2_ with shaking. Horse serum was used over lysed horse blood to enable turbidity readings at 600 nm. Quality control MIC ranges according to CLSI standards are as outlined in Table S3.

**Fig 2:**
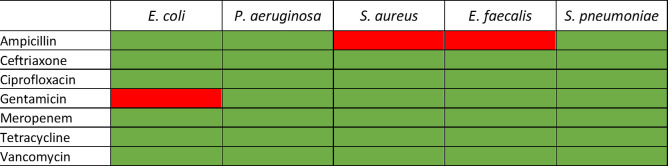
Validation of NYCIII as a growth medium for AST evaluation. MICs were determined for test microbes in both the standard medium and NYCIII and compared. If both test conditions yielded MICs that were within QC range, then the cell is shaded green. If MICs were outside QC range, then the cell is shaded red. For actual MIC values, see Table S3.

With the QC MIC ranges as a reference, these microbes then were grown in their CLSI standard medium and compared to growth in the experimental NYCIII medium in the presence of antibiotics (Fig. 2; Table S4). Observed MIC values of reference microbes agreed for both media for all antibiotics tested with three exceptions. First, *E. faecalis* ATCC29212 exhibited susceptibility to ampicillin in the standard CAMHB condition at 0.25 µg/mL, below the 0.5 µg/mL QC range; however, the same strain behaved within the QC range when grown in NYCIII. Second, *E. coli* ATCC25922 exhibited increased resistance beyond the QC range for gentamicin when grown in NYCIII (>1 µg/mL). Similarly, *S. aureus* ATCC29213 exhibited increased resistance to ampicillin when grown in NYCIII (>2 µg/mL).

### Determination of MIC of fastidious urinary isolates

Utilizing this method of AST, we evaluated the MICs of fastidious clinical isolates of the human urinary tract. We first selected species that were capable of growing in ambient air supplemented with 5% CO_2_. These included *Actinomyces turicensis*, *Actinotignum timonense*, *Aerococcus urinae* complex, *Corynebacterium aurimucosum*, *Streptococcus agalactiae*, *Streptococcus anginosus*, and two coagulases negative *Staphylococci* (CoNS): *Staphylococcus lugdunensis* and *Staphylococcus simulans* (Table 2; Table S5). For each species, at least four different strains were tested in duplicate.

**TABLE 2:** Summarized susceptibility ranges of selected fastidious urinary bacteria in NYCIII

Ampicillin	No.^*a*^	>85 µg/mL	85 µg/mL	8.5 µg/mL	0.85 µg/mL	0.085 µg/mL
*Actinomyces turicensis*	4			1	2	1
*Actinotignum timonense*	5	2	2	1		
*Aerococcus urinae* complex*^b^*	5			1	3	1
*Corynebacterium aurimucosum*	5		1	4		
*Staphylococcus lugdunensis*	8		4	4		
*Staphylococcus simulans*	5		1	1	3	
*Streptococcus agalactiae*	5	1			3	1
*Streptococcus anginosus*	6		2	4		

Ciprofloxacin	No.	>20 µg/mL	20 µg/mL	2 µg/mL	0.2 µg/mL	0.02 µg/mL
*Actinomyces turicensis*	4	4				
*Actinotignum timonense*	5	2	2	1		
*Aerococcus urinae* complex*^b^*	5	1	3	1		
*Corynebacterium aurimucosum*	5	4	1			
*Staphylococcus lugdunensis*	8			2	6	
*Staphylococcus simulans*	5	1	2	1	1	
*Streptococcus agalactiae*	5		5			
*Streptococcus anginosus*	6	4	2			

Ceftriaxone	No.	>64 µg/mL	64 µg/mL	6.4 µg/mL	0.64 µg/mL	0.064 µg/mL
*Actinomyces turicensis*	4				2	2
*Actinotignum timonense*	5			1	3	1
*Aerococcus urinae* complex*^b^*	5			1	3	1
*Corynebacterium aurimucosum*	5		1	4		
*Staphylococcus lugdunensis*	8			8		
*Staphylococcus simulans*	5		1	4		
*Streptococcus agalactiae*	5			4	1	
*Streptococcus anginosus*	6			1	5	

Gentamicin	No.	>17 µg/mL	17 µg/mL	1.7 µg/mL	0.17 µg/mL	0.017 µg/mL
*Actinomyces turicensis*	4	4				
*Actinotignum timonense*	5	1	4			
*Aerococcus urinae* complex*^b^*	5	5				
*Corynebacterium aurimucosum*	5	1	2	2		
*Staphylococcus lugdunensis*	8		5	3		
*Staphylococcus simulans*	5		4	1		
*Streptococcus agalactiae*	5	5				
*Streptococcus anginosus*	6	1	5			

Meropenem	No.	>91 µg/mL	91 µg/mL	9.1 µg/mL	0.91 µg/mL	0.091 µg/mL
*Actinomyces turicensis*	4		2	1	1	
*Actinotignum timonense*	5					5
*Aerococcus urinae* complex*^b^*	5					5
*Corynebacterium aurimucosum*	5		3	2		
*Staphylococcus lugdunensis*	8	1		1	1	5
*Staphylococcus simulans*	5					5
*Streptococcus agalactiae*	5					5
*Streptococcus anginosus*	6				3	3

Tetracycline	No.	>32 µg/mL	32 µg/mL	3.2 µg/mL	0.32 µg/mL	0.032 µg/mL
*Actinomyces turicensis*	4		2	2		
*Actinotignum timonense*	5		2	2	1	
*Aerococcus urinae* complex*^b^*	5		1	1	3	
*Corynebacterium aurimucosum*	5		4		1	
*Staphylococcus lugdunensis*	8			1	6	1
*Staphylococcus simulans*	5			3	2	
*Streptococcus agalactiae*	5		4	1		
*Streptococcus anginosus*	6	1	4	1		

Trimethoprim	No.	>64 µg/mL	64 µg/mL	6.4 µg/mL	0.64 µg/mL	0.064 µg/mL
*Actinomyces turicensis*	4	3	1			
*Actinotignum timonense*	5	5				
*Aerococcus urinae* complex*^b^*	5		5			
*Corynebacterium aurimucosum*	5		3	2		
*Staphylococcus lugdunensis*	8		8			
*Staphylococcus simulans*	5	5				
*Streptococcus agalactiae*	5	2	3			
*Streptococcus anginosus*	6		2	4		

Vancomycin	No.	>41 µg/mL	41 µg/mL	4.1 µg/mL	0.41 µg/mL	0.041 µg/mL
*Actinomyces turicensis*	4			4		
*Actinotignum timonense*	5			5		
*Aerococcus urinae* complex*^b^*	5		1	4		
*Corynebacterium aurimucosum*	5			5		
*Staphylococcus lugdunensis*	8		8			
*Staphylococcus simulans*	5		5			
*Streptococcus agalactiae*	5	1		4		
*Streptococcus anginosus*	6			6		

^*a*^ "No.” refers to the number of isolates tested per species.

^*b*^ *Aerococcus urinae* has recently been revealed to be a complex of at least four species: *A. urinae*, *A. tenax*, *A. mictus*, and *A. loyolae* (20). The type strain of each species was tested here.

### Evaluation of urinary anaerobes

Isolation and growth of anaerobic bacteria from the urinary tract are not common as the equipment to foster anaerobic environments is rarely found in clinical settings. However, their presence often can be detected via enhanced culture methods, such as EQUC, and metagenomic sequencing techniques, such as via 16S rRNA gene and shotgun metagenomic sequencing. Thus, we determined whether our methodology also could be used to evaluate AS of these microbes. These include *Anaerococcus* sp*.*, *Gardnerella vaginalis*, *Lactobacillus crispatus*, *Lactobacillus iners*, *Lactobacillus jensenii*, *Lactobacillus gasseri*, *Peptoniphilus harei*, and *Propionimicrobium lymphophilum*. As a proof of concept, we evaluated susceptibility to tetracycline in an anaerobic environment with incubation for 72 hours (Table 3).

**TABLE 3:** Summarized susceptibility ranges for select fastidious organisms in anaerobic conditions to tetracycline in NYCIII

Tetracycline	No.^*a*^	>32 µg/mL	32 µg/mL	3.2 µg/mL	0.32 µg/mL	0.032 µg/mL
*Anaerococcus* sp.	3		1		2	
*Gardnerella vaginalis*	4		2		2	
*Lactobacillus crispatus*	4	1	3			
*Lactobacillus iners*	3	2	1			
*Lactobacillus jensenii*	3		3	1		
*Lactobacillus gasseri*	4	1	1	2		
*Peptoniphilus harei*	4		1	3		
*Propionimicrobium lymphophilum*	2			1	1	

^*a*^ "No.” refers to the number of isolates tested per species.

## DISCUSSION

The human lower urinary tract features a variety of ecological niches that can foster the growth of a wide diversity of bacterial species. Simulating these diverse environmental conditions outside of the human body to study these bacteria remains one of the greatest challenges for microbial ecologists, let alone the average clinician. As such, using components that most resemble the native environment for these microbes while balancing accessibility and costs for the clinical lab is always an ongoing struggle. In this study, we evaluated the usage of NYCIII media, originally developed for isolation of *Neisseria* species, as a potential harmonized medium to test AST in urinary isolates.

While there exists a variety of tests specifically developed to evaluate AST in certain species, no generalized testing medium exists that can evaluate non-fastidious, fastidious, and anaerobic bacteria of the human urinary tract. The major advantage of such a harmonized medium would be the ability to conduct a direct comparison of values between species without having to account for differences in media or methodologies. Furthermore, a harmonized medium can allow for polymicrobial AST as it has been demonstrated that isolates of a polymicrobial culture behave differently together than when tested in isolation (21).

While MHB remains the standard for most bacterial AST as recommended by CLSI and EUCAST guidelines, the medium does not support the growth of many fastidious bacteria and most anaerobic bacteria of the urinary tract. Only with supplementation with lysed horse blood is growth rescued. However, the blood renders the medium opaque preventing turbidity measurements of bacterial growth via spectrometry. Thus, we evaluated NYCIII, a transparent medium using horse serum instead of horse blood, to evaluate AST. From our results, we demonstrate that NYCIII can support the growth of both fastidious and non-fastidious bacteria to levels similar to that of CAMHB-LHB. When testing AS ranges for QC standard strains, NYCIII can meet similar values, indicating direct translatability between standard MHB and NYCIII MIC values. Thus, NYCIII may be a feasible medium to conduct AST in a 96-well format via plate-reader spectrometry. We propose further testing for this medium with the goal toward standardization, such that AST guidelines may be established for the many emerging uropathogens.

Indeed, with this medium, many urinary strains can be simultaneously compared at once to evaluate relative susceptibility. While screening selected specimens, we were able to identify several isolates that exhibited susceptibilities beyond the tested range, potentially indicating resistance. It is important to note that no resistance cutoffs have been established for these bacteria and, thus, the prevalence of resistance remains unknown.

As long as these emerging uropathogens remain uncharacterized, physicians will continue to prescribe antibiotics without guidance. The implications of these are several-fold including prolonged therapies, over-prescription, and poor antibiotic stewardship. Accordingly, urinary microbes that are not under active surveillance are potentially harboring antibiotic resistances. Even non-pathogenic bacteria can serve as reservoirs of resistance for pathogenic bacteria, such as through horizontal gene transfer (22). As such, establishing AST patterns in newly identified bacteria, and especially for emerging uropathogens, may be easier to conduct with our proposed harmonized methodology. Additionally, it may be of value to conduct AST of commensals, particularly bacteria species considered protective against disease, to establish their vulnerability to antibiotics.

As for limitations, only a small number of strains were tested for each species in this study, and thus, these MICs should not be used as reference values for clinical purposes. Rather, values found in this study should be interpreted as an evaluation of the feasibility of employing NYCIII as a valid AST methodology such that more in-depth studies may be conducted in the future. Furthermore, it may be important to take MICs of sulfonamides and trimethoprim when using NYCIII with a grain of salt. Media that contain relatively high concentrations of thymidine are known to interfere with the inhibitory effect of sulfonamides and trimethoprim (23). However, media that use horse blood have an advantage of containing thymidine phosphorylase borne from lysed horse erythrocytes (24). This enzyme converts thymidine to inactive thymine, allowing both antibiotics to work uninhibited. Since NYCIII replaces lysed horse blood with horse serum, this advantage has been lost.

### Conclusion

In this study, we evaluate the use of a proposed harmonized medium, NYCIII, in conducting AST of clinical urinary isolates. The medium offers the ability to conduct AST in a 96-well format, allowing for the rapid screening of relative AST of both fastidious and anaerobic urinary bacteria. Further standardization of this medium may greatly improve the evaluation of resistance of emerging uropathogens in the clinical setting.

## AFTER EPUB

*[This article was published on 6 May 2024 with an error in the abstract. The error was corrected in the current version, posted on 9 May 2024.]*

## SUPPLEMENTAL MATERIAL

Supplemental tables spectrum.00314-24-s0001.pdf Tables S1-S5.

ASM does not own the copyrights to Supplemental Material that may be linked to, or accessed through, an article. The authors have granted ASM a non-exclusive, world-wide license to publish the Supplemental Material files. Please contact the corresponding author directly for reuse.
