# Supplementary material for: Microbroth dilution method for antibiotic susceptibility testing of fastidious and anaerobic bacteria of the urinary microbiome
Source: Microbiol Spectr. 2024 May 6;12(6):e00314-24. doi: 10.1128/spectrum.00314-24 (PMC11237461; doi:10.1128/spectrum.00314-24)
Supplement: Supplemental tables — Tables S1-S5. [file spectrum.00314-24-s0001.pdf]

Supplemental Material

Supplemental Table S1 – Strain List

Supplemental Table S2 – Complete Materials List

Supplemental Table S3 – Quality Control Reference MIC ranges

Supplemental Table S4 – MIC Comparison of QC Strains in Standard vs NYCIII Media

Supplemental Table S5 – Determination of MIC for Fastidious and Anaerobic Urinary Isolates in NYCIII

Supplemental Table S1. Strain List

Strain Name	Species Identity
UMB8616	*Streptococcus anginosus*
UMB7768	*Streptococcus anginosus*
UMB7052	*Streptococcus anginosus*
UMB1353	*Streptococcus anginosus*
UMB0141	*Streptococcus anginosus*
UMB4708	*Streptococcus anginosus*
UMB8037	*Actinotignum timonense*
UMB8968	*Actinotignum timonense*
UMB9950	*Actinotignum timonense*
UMB8705	*Actinotignum timonense*
UMB9903	*Actinotignum timonense*
ATCC TSD-300T	*Aerococcus loyolae*
ATCC TSD-301T	*Aerococcus mictus*
ATCC TSD-302T	*Aerococcus tenax*
UMB0722	*Aerococcus urinae*
ATCC 51268T	*Aerococcus urinae*
UMB7318	*Staphylococcus lugdunensis*
UMB7425	*Staphylococcus lugdunensis*
UMB7854	*Staphylococcus lugdunensis*
UMB7994	*Staphylococcus lugdunensis*
UMB12897	*Staphylococcus lugdunensis*
UMB13010	*Staphylococcus lugdunensis*
UMB13042	*Staphylococcus lugdunensis*
UMB13101	*Staphylococcus lugdunensis*
UMB0529	*Streptococcus agalactiae*
UMB3640	*Streptococcus agalactiae*
UMB4546	*Streptococcus agalactiae*
UMB5285	*Streptococcus agalactiae*
UMB11507	*Streptococcus agalactiae*
UMB0230	*Staphylococcus simulans*
UMB2030	*Staphylococcus simulans*
UMB6618	*Staphylococcus simulans*
UMB8267	*Staphylococcus simulans*
UMB10066	*Staphylococcus simulans*
UMB0033	*Lactobacillus iners*

UMB1051	*Lactobacillus iners*
UMB6790	*Lactobacillus iners*
UMB0821	*Lactobacillus crispatus*
UMB4833	*Lactobacillus crispatus*
UMB12449	*Lactobacillus crispatus*
UMB13119	*Lactobacillus crispatus*
UMB3077	*Lactobacillus gasseri*
UMB8100	*Lactobacillus gasseri*
UMB9792	*Lactobacillus gasseri*
UMB10322	*Lactobacillus gasseri*
UMB7766	*Lactobacillus jensenii*
UMB1186	*Lactobacillus jensenii*
UMB13194	*Lactobacillus jensenii*
UMB7398	*Corynebacterium aurimucosum*
**ATCC 25922**	*Escherichia coli*
**ATCC 27853**	*Pseudomonas aeruginosa*
**ATCC 29213**	*Staphylococcus aureus*
**ATCC 29212**	*Enterococcus faecalis*
**ATCC 49619**	*Streptococcus pneumoniae*
UMB0065	*Peptoniphilus harei*
UMB8120	*Peptoniphilus harei*
UMB9796	*Peptoniphilus harei*
UMB13140	*Peptoniphilus harei*
UMB0386	*Gardnerella vaginalis*
UMB0411	*Gardnerella vaginalis*
UMB1190	*Gardnerella vaginalis*
UMB0540	*Gardnerella vaginalis*
UMB0204	*Anaerococcus vaginalis*
UMB9705	*Anaerococcus hydrogenalis*
UMB13234	*Anaerococcus hydrogenalis*
UMB11620	*Propionibacterium lymphophilum*
UMB9327	*Propionimicrobium lymphophilum*

CLSI reference strains are in bold

Supplemental Table S2. Complete Materials List

Item	Vendor / Part#
Mueller Hinton Broth	Sigma / 70192
Ampicillin sodium salt	Sigma / A-9518
Ceftriaxone disodium salt	Fisher / C22265G
Ciprofloxacin	Fisher / AC449620050
Gentamicin sulfate	Fisher / AC455310050
Meropenem trihydrate	Fisher / M22791G
Tetracycline	Fisher / AAJ6171406
Vancomycin hydrochloride	Fisher / J62790
Cytiva HyClone™ HEPES Solution	Fisher / SH3023701
GibcoTM Bacto™ Proeose Peptone No. 3	Fisher / DF0122-07-6
Cytiva HyClone™ Newborn Bovine Calf Serum, Heat Inactivated	Fisher/ SH3011803HI
Horse Serum USA Origin, Heat Inactivated	Sigma / H1270-1L
Yeast Extract	Fisher / BP1422-500
D (+) Glucose	Sigma / G8220-1kg
Sodium Chloride (≥99.5%)	Sigma / S7653
Cyrosaver™ Brucella w/ Glycerol	Hardy Diagnostics / CS100BNB
96 Well Tissue Culture Plate with Lid, Flat bottom Polystyrene	DotSci / 667196
500mL vacuum filtration funnel .22um	VWR / 10040-468
Lysed Horse Blood	LAMPIRE / 50-414-843
Mueller Hinton Broth 2 (Cation adjusted)	Sigma / 90922
TSA with 5% Sheep Blood plated media	Fisher / B21261X

Supplemental Table S3. Quality Control Reference MIC ranges

	ATCC25922 *E. coli*	ATCC27853 *P. aeruginosa*	ATCC29213 *S. aureus*	ATCC29212 *E. faecalis*	ATCC49619 *S. pneumoniae*
**Antibiotic**					
Ampicillin	2-8	N/A	0.5-2	0.5–2	0.06–0.25
Ceftriaxone	0.03–0.12	8–64	1–8	N/A	0.03–0.12
Ciprofloxacin	0.004–0.016	0.12–1	0.12–0.5	0.25–2	N/A
Gentamicin	0.25–1	0.5–2	0.12–1	4–16	N/A
Meropenem	0.008–0.06	0.12–1	0.03–0.12	2–8	0.03–0.25
Tetracycline	0.5–2	8–32	0.12–1	8–32	0.06–0.5
Vancomycin	N/A	N/A	0.5-2	1-4	0.12–0.5

Values in µg/mL

Supplemental Table S4. MIC Comparison of QC Strains in Standard vs NYCIII Media

Strain	Species	Antibiotic	MIC QC range (µg/mL)	MHB MIC (µg/mL)	NYCIII MIC (µg/mL)	MHB in range?	NYCIII in range?
**ATCC 25922**	*E. coli*	AMP	2-8	4	4	y	y
**ATCC 25922**	*E. coli*	CEFT	0.03–0.12	0.06	0.03	y	y
**ATCC 25922**	*E. coli*	CIP	0.004–0.016	0.0008	0.016	y	y
**ATCC 25922**	*E. coli*	GENT	0.25–1	1	>1	y	n
**ATCC 25922**	*E. coli*	MER	0.008–0.06	0.03	0.03	y	y
**ATCC 25922**	*E. coli*	TET	0.5–2	1	1	y	y
**ATCC 25922**	*E. coli*	VANC	N/A	N/A	N/A		
**ATCC 27853**	*P. aeruginosa*	AMP	N/A	N/A	N/A		
**ATCC 27853**	*P. aeruginosa*	CEFT	8–64	8	32	y	y
**ATCC 27853**	*P. aeruginosa*	CIP	0.12–1	0.25	0.5	y	y
**ATCC 27853**	*P. aeruginosa*	GENT	0.5–2	1	2	y	y
**ATCC 27853**	*P. aeruginosa*	MER	0.12–1	0.25	0.5	y	y
**ATCC 27853**	*P. aeruginosa*	TET	8–32	8	16	y	y
**ATCC 27853**	*P. aeruginosa*	VANC	N/A	N/A	N/A		
**ATCC 29213**	*S. aureus*	AMP	0.5-2	2	>2	y	n
**ATCC 29213**	*S. aureus*	CEFT	1–8	4	2	y	y
**ATCC 29213**	*S. aureus*	CIP	0.12–0.5	0.25	0.25	y	y
**ATCC 29213**	*S. aureus*	GENT	0.12–1	1	0.5	y	y
**ATCC 29213**	*S. aureus*	MER	0.03–0.12	0.06	0.06	y	y
**ATCC 29213**	*S. aureus*	TET	0.12–1	1	0.25	y	y
**ATCC 29213**	*S. aureus*	VANC	0.5-2	1	2	y	y
**ATCC 29212**	*E. faecalis*	AMP	0.5–2	0.25	1	n	y
**ATCC 29212**	*E. faecalis*	CEFT	N/A	N/A	N/A		
**ATCC 29212**	*E. faecalis*	CIP	0.25–2	0.5	1	y	y
**ATCC 29212**	*E. faecalis*	GENT	4–16	8	8	y	y
**ATCC 29212**	*E. faecalis*	MER	2–8	2	2	y	y
**ATCC 29212**	*E. faecalis*	TET	8–32	16	16	y	y

**ATCC 29212**	*E. faecalis*	VANC	1-4	1	4	y	y
**ATCC 49619**	*S. pneumoniae*	AMP	.06-.25	0.125	0.125	y	y
**ATCC 49619**	*S. pneumoniae*	CEFT	.03-.12	0.06	0.03	y	y
**ATCC 49619**	*S. pneumoniae*	CIP	N/A	N/A	N/A		
**ATCC 49619**	*S. pneumoniae*	GENT	N/A	N/A	N/A		
**ATCC 49619**	*S. pneumoniae*	MER	.03-.25	0.0625	0.03	y	y
**ATCC 49619**	*S. pneumoniae*	TET	.06-.5	0.25	0.25	y	y
**ATCC 49619**	*S. pneumoniae*	TRI	.12-1	0.5	0.5	y	y
**ATCC 49619**	*S. pneumoniae*	VANC	.12-.5	0.5	0.5	y	y

MIC QC ranges retrieved from CLSI M100 5A-2 and 5B. AMP = Ampicillin, CEFT = Ceftriaxone, CIP = Ciprofloxacin, GENT = Gentamicin, MER = Meropenem, TET = Tetracycline, TRI = Trimethoprim, VANC = Vancomycin. All tests were conducted in duplicate.

Supplemental Table S5. Determination of MIC for Fastidious and Anaerobic Urinary Isolates in NYCIII

Strain	Species	MER	VANC	GENT	CIP	AMP	TRI	TET	CEFT
UMB8616	*Streptococcus anginosus*	0.091	4.1	17	200	8.5	6.4	32	0.64
UMB7768	*Streptococcus anginosus*	0.91	4.1	170	200	85	64	32	6.4
UMB7052	*Streptococcus anginosus*	0.091	4.1	17	20	8.5	6.4	>32	0.64
UMB1353	*Streptococcus anginosus*	0.91	4.1	17	20	8.5	64	3.2	0.64
UMB0141	*Streptococcus anginosus*	0.91	4.1	17	200	8.5	6.4	32	0.64
UMB4708	*Streptococcus anginosus*	0.091	4.1	17	200	85	6.4	32	0.64
UMB8037	*Actinotignum timonense*	0.091	4.1	17	2	>85	64	0.32	0.064
UMB8968	*Actinotignum timonense*	0.091	4.1	17	20	>85	64	32	0.64
UMB9950	*Actinotignum timonense*	0.091	4.1	17	20	8.5	64	3.2	0.64
UMB8705	*Actinotignum timonense*	0.091	4.1	170	200	85	64	32	6.4
UMB9903	*Actinotignum timonense*	0.091	4.1	17	200	85	64	3.2	0.64
UMB0080	*Aerococcus loyolae*	0.091	4.1	170	20	0.85	64	0.32	0.064
UMB3440	*Aerococcus mictus*	0.091	4.1	170	200	0.85	64	0.32	0.64
UMB3669	*Aerococcus tenax*	0.091	4.1	170	20	8.5	64	3.2	6.4
UMB0722	*Aerococcus urinae*	0.091	41	170	20	0.85	64	32	0.64
ATCC51268	*Aerococcus urinae*	0.091	4.1	170	2	0.085	64	0.32	0.64
UMB7318	*Staphylococcus lugdunensis*	0.91	41	17	0.2	85	64	3.2	6.4
UMB7425	*Staphylococcus lugdunensis*	0.091	41	1.7	0.2	85	64	0.032	6.4
UMB7854	*Staphylococcus lugdunensis*	0.091	41	17	0.2	8.5	64	0.32	6.4
UMB7994	*Staphylococcus lugdunensis*	9.1	41	17	0.2	85	64	0.32	6.4
UMB12897	*Staphylococcus lugdunensis*	0.091	41	17	0.2	85	64	0.32	6.4
UMB13010	*Staphylococcus lugdunensis*	>91	41	1.7	0.2	8.5	64	0.32	6.4
UMB13042	*Staphylococcus lugdunensis*	0.091	41	17	2	8.5	64	0.32	6.4
UMB13101	*Staphylococcus lugdunensis*	0.091	41	1.7	2	8.5	64	0.32	6.4
UMB0529	*Streptococcus agalactiae*	0.091	4.1	170	20	0.085	64	32	6.4
UMB3640	*Streptococcus agalactiae*	0.091	4.1	170	20	0.85	64	32	6.4
UMB4546	*Streptococcus agalactiae*	0.0091	4.1	170	20	0.85	64	32	0.64
UMB5285	*Streptococcus agalactiae*	0.091	>41	>170	20	>85	6.4	3.2	6.4
UMB11507	*Streptococcus agalactiae*	0.091	4.1	170	20	0.85	64	32	6.4
UMB0230	*Staphylococcus simulans*	0.091	41	1.7	2	0.85	64	0.32	6.4
UMB2030	*Staphylococcus simulans*	0.091	41	17	20	0.85	64	3.2	6.4
UMB6618	*Staphylococcus simulans*	0.091	41	17	20	0.85	64	3.2	6.4
UMB8267	*Staphylococcus simulans*	0.91	41	17	200	85	64	3.2	64
UMB10066	*Staphylococcus simulans*	0.091	41	17	0.2	8.5	64	0.32	6.4
UMB0821	*Lactobacillus crispatus*	91	4.1	170	200	0.85	64	>32	64
UMB4833	*Lactobacillus crispatus*							32	
UMB12449	*Lactobacillus crispatus*							32	

UMB13119	*Lactobacillus crispatus*							32	
UMB0033	*Lactobacillus iners*							32	
UMB1051	*Lactobacillus iners*							>32	
UMB6790	*Lactobacillus iners*							>32	
UMB3077	*Lactobacillus gasseri*							>32	
UMB8100	*Lactobacillus gasseri*							3.2	
UMB9792	*Lactobacillus gasseri*							3.2	
UMB10322	*Lactobacillus gasseri*							32	
UMB1186	*Lactobacillus jensenii*							32	
UMB7766	*Lactobacillus jensenii*							32	
UMB13194	*Lactobacillus jensenii*							3.2	
UMB0204	*Anaerococcus vaginalis*							32	
UMB9705	*Anaerococcus hydrogenalis*							0.32	
UMB13234	*Anaerococcus hydrogenalis*							0.32	
UMB0386	*Gardnerella vaginalis*							32	
UMB0411	*Gardnerella vaginalis*							32	
UMB1190	*Gardnerella vaginalis*							0.32	
UMB0540	*Gardnerella vaginalis*							0.32	
UMB0065	*Peptoniphilus harei*							32	
UMB8120	*Peptoniphilus harei*							3.2	
UMB9796	*Peptoniphilus harei*							3.2	
UMB13140	*Peptoniphilus harei*							3.2	
UMB11620	*Propinonibacterium lymphophilum*							3.2	
UMB9327	*Propinonibacterium lymphophilum*							3.2	

AMP = Ampicillin, CEFT = Ceftriaxone, CIP = Ciprofloxacin, GENT = Gentamicin, MER = Meropenem,

TET = Tetracycline, TRI = Trimethoprim, VANC = Vancomycin
